# Durvalumab-induced myocarditis, myositis, and myasthenia gravis: a case report

**DOI:** 10.1186/s13256-021-02858-7

**Published:** 2021-05-31

**Authors:** Jason Cham, Daniel Ng, Laura Nicholson

**Affiliations:** Department of Internal Medicine, Scripps Clinic/Scripps Green Hospital, 10666 N Torrey Pines Rd 403C, La Jolla, CA 92037-1027 USA

**Keywords:** Checkpoint inhibitors, Immunotherapy, Adverse effects, Myocarditis, Myositis, Myasthenia gravis, Non-small cell lung cancer

## Abstract

**Background:**

Immune checkpoint inhibitors are effective therapies for a wide range of malignancies. Their increased use has led to a wide range of immune-related adverse effects including skin, gastrointestinal, pulmonary, endocrine, cardiac, and neurologic complications.

**Case presentation:**

We present the case of a 72-year-old Caucasian man with non-small cell lung cancer who was admitted for dyspnea after two cycles of durvalumab. He was found to have significantly elevated levels of serum creatinine kinase and troponin with a negative cardiac catheterization. During his hospitalization, he developed progressive dyspnea and new-onset axial weakness, ultimately leading to the diagnosis of durvalumab-induced myocarditis, myasthenia gravis, and myositis.

**Conclusion:**

This is, to our knowledge, the first reported case of anti-programmed cell death ligand 1-induced combination of myocarditis, myasthenia gravis, and myositis. While the use of immunologic agents has resulted in overall improved cancer outcomes, their increased use has led to a vast spectrum of immune-related adverse effects. We review the diagnostic workup and management of patients with these immune-related adverse effects, underscoring the importance of early identification given the potential for rapid deterioration.

## Background

Durvalumab (MedImmune/AstraZeneca) is a human immunoglobulin G1 kappa monoclonal antibody directed against programmed cell death ligand 1 (PD-L1). It has been demonstrated to have significant antitumor activity and is FDA-approved for use in non-small cell lung cancer and urothelial carcinoma. In March 2020, approval was added for durvalumab in combination with etoposide and either carboplatin or cisplatin as a first-line treatment of patients with extensive small cell lung cancer [1]. While immune checkpoint inhibitors (ICI) are now a mainstay of treatment for numerous malignancies, they are also associated with immune-related adverse effects (irAE). The most common irAE for durvalumab are immune-mediated dermatologic reactions (1.6%), colitis (1.6%), hepatitis (1%), nephritis (0.3%), and endocrinopathies including hypothyroidism (7.3%) and hyperthyroidism (1.4%), adrenal insufficiency (0.4%), type 1 diabetes mellitus (< 0.1%), and hypopituitarism/hypophysitis (< 0.1%). [1] Here, we present a patient who developed a rare combination of adverse effects: myocarditis, myositis, and myasthenia gravis, as a result of PD-L1 inhibition with durvalumab. 

## Case presentation

A 72-year-old Caucasian man with a history of prostate cancer, central serous retinopathy, obstructive sleep apnea, type 2 diabetes mellitus, hypertension, and mild chronic obstructive pulmonary disease but no known coronary artery disease or history of neurologic disorders was first diagnosed with left lower lobe non-small cell lung cancer (NSCLC) in 2013, for which he underwent a left lower lobectomy that same year. On surveillance imaging in 2014, he was noted to have a right lower lobe nodule and was diagnosed with a second primary NSCLC, for which he underwent stereotactic body radiation therapy (SBRT). In 2017, he was noted to have an enlarging left upper lobe nodule that was treated with SBRT. Surveillance CT imaging in September 2018 was significant for an enlarging right upper lobe nodule with right paratracheal lymph node involvement. Subsequent positron emission tomography (PET)-CT revealed several FDG-avid mediastinal and right hilar nodes concerning for malignancy. Metastatic bronchogenic adenocarcinoma was confirmed via endobronchial ultrasound with transbronchial needle aspiration, with pathology showing 5/5 positive lymph nodes. PD-L1 immunohistochemistry 22C3 (Keytruda) testing of the lymph node revealed high PD-L1 expression with 80% tumor proportion score. MRI of the brain was negative for intracranial metastatic disease. He was diagnosed with TxN3M0 stage IIIB adenocarcinoma and was treated with six cycles of weekly cisplatin and SBRT. He then initiated adjuvant durvalumab for maintenance therapy, receiving two cycles (10 mg/kg) on treatment days 0 and 15. Our patient subsequently presented to the emergency department on day 18 with a 4-day history of shortness of breath, weakness, and chest pressure. Workup on admission was significant for lateral lead ST elevations with an initial troponin of 7.1 ng/dL. He emergently underwent a left heart catheterization, which was negative for obstructive coronary artery disease. Transthoracic echocardiogram was significant for a normal ejection fraction with mild left ventricular concentric hypertrophy.

Admission diagnosis was myopericarditis of unclear etiology. On hospital day 4, he was noted to have persistently elevated levels of serum troponin, peaking at 12 ng/mL. His levels of serum creatinine kinase (CK) were elevated as well, peaking at 9262 U/L. He had persistent mild hepatocellular transaminitis (peak AST 72, ALT 531). The use of corticosteroids was discussed with the patient owing to concern for irAE; however the patient declined on the basis of his central serous retinopathy. He was noted to have dysphagia on admission with regurgitation of solids and liquids. A barium swallow study and esophagogastroduodenoscopy on hospital day 3 did not reveal any structural abnormalities. On hospital day 9, the patient developed progressive axial weakness, with increasing difficulty holding his head upright while seated. Neurology was consulted, who had a high concern for myasthenic crisis, which was subsequently confirmed by decreased negative inspiratory forces and elevated acetylcholine receptor binding and blocking antibodies. Due to his declining respiratory status, he was transferred to the intensive care unit (ICU) and intubated on the same day for airway protection. MRI of the brain performed on hospital day 10 was significant for 12 new metastatic lesions with surrounding vasogenic edema. The patient was started on high-dose corticosteroids at 1 mg/kg/day and underwent plasmapheresis on hospital day 10, completing 5 rounds. Unfortunately, he was unable to be weaned from mechanical ventilation and required tracheostomy placement as well as percutaneous endoscopic gastrostomy nutrition.

After multiple goals-of-care discussions, the patient was transferred from the ICU to a long-term acute care facility owing to mechanical ventilation dependence, on hospital day 36. Diagnoses of myopericarditis, myositis, and myasthenic crisis were attributed to immune-mediated response to durvalumab.

## Discussion and conclusions

The differential diagnosis for dyspnea and weakness is broad, but they may be the presenting symptoms for irAE in a patient on immunotherapy. This report presents an individual who developed a rare combination of adverse effects: myocarditis, myositis, and myasthenia gravis (MG) as a result of PD-L1 inhibition with durvalumab.

ICI-related myocarditis is a rare irAE with a reported incidence of 0.04–1.14% but is associated with a mortality of 25–50% [2]. Female patients, patients > 75 years old, and patients with concomitant use of other immune checkpoint inhibitors have been found to have significantly increased risk for myocarditis [3]. There is a wide spectrum of severity, ranging from asymptomatic elevation in cardiac enzyme levels to end organ dysfunction as categorized by the American Society of Clinical Oncology. This patient presented with ST elevation and marked elevation in cardiac enzyme levels, but was found to have no obstructive coronary artery disease, leading to the diagnosis of likely ICI-related myocarditis. First-line treatment relies on high-dose corticosteroids and immediate discontinuation of the offending agent. Second-line treatment options remain unproven, but include intravenous immunoglobulin, mycophenolate, infliximab, alemtuzumab, and abatacept [4]. Our patient had a relative contraindication to steroids due to his central serous retinopathy, but given his severe symptoms was ultimately treated with high-dose steroids and plasmapheresis.

Paraneoplastic neurologic syndromes such as Lambert-Eaton myasthenic syndrome (LEMS) have been clearly established to be associated with lung carcinoma. LEMS was considered upon development of respiratory failure; however, the patient had negative voltage-gated calcium channel antibodies, which would be inconsistent with the diagnosis. Myasthenia gravis as a paraneoplastic syndrome in primary lung carcinomas has not been clearly described in the literature; as such, it was thought that our patient’s myasthenic crisis arose from the ICI [5]. ICI-related MG is a rare irAE with a reported incidence of less than 1%. ICI-related MG often occurs between 2 and 6 weeks of initiation of checkpoint inhibitors. Thus, given the time course of symptoms, ICI-related MG is the more likely diagnosis. Compared with classical myasthenia gravis, which has a bimodal age distribution and female predominance, MG associated with immune checkpoint inhibitors seems to occur mostly in men, at a relatively older onset [6]. Two pathogenic autoantibodies are present in the majority of MG patients: 85% have anti-acetylcholine receptor autoantibodies (AchR), and 8% have anti-muscle-specific tyrosine kinase (MuSK) autoantibodies [7]. Our patient was found to have anti-AchR antibodies. In a large retrospective study of immunotherapy-induced MG by the anti-PD1 nivolumab, 8 of 12 patients developed severe symptoms including myasthenic crisis [8]. Onset of MG occurred early in their treatment course (within one or two treatments), and patients developed rapid progression, consistent with the clinical course of our patient. Prompt identification of this rare adverse event is imperative as respiratory support either through noninvasive positive pressure ventilation or mechanical ventilation may be necessary, given the potential for acute respiratory failure secondary to progressive MG. In patients with mild symptoms, pyridostigmine can be used, but in patients with severe symptoms and rapid progression, immunosuppression with intravenous methylprednisolone is required [4]. There is some evidence suggesting that intravenous immunoglobulin (IVIG) and plasma exchange as a first-line therapy may have better outcomes compared with steroids alone [9].

Finally, myositis can co-occur in patients who develop MG. In most reported cases, CK was significantly elevated, with reported levels ranging from 1200 to 8729 IU/L [10]. While muscle biopsy was not obtained in our patient and in most reported cases, the presence of elevated CK along with transaminases likely reflects muscle damage rather than hepatic injury as our patient had normal bilirubin and INR and unremarkable liver imaging. In an observational, retrospective study of patients with idiopathic MG and inflammatory myopathies, seven of eight patients had antistriational autoantibodies, and three of these patients also developed myocarditis [11]. This suggests there may be autoimmune targets with similar epitopes on heart and skeletal muscles. ICI-induced MG with antistriational antibodies has been associated with rapid progression and more severe outcomes, so early antibody screening may assist in prompt detection and prognostication of severe irAEs [11].

The combination of myocarditis, MG, and myositis has been reported with different ICI, including ipilimumab and nivolumab [12]. Myocarditis has been previously reported in patients treated with durvalumab, but this, to our knowledge, is the first reported case of anti-PD-L1-induced combination of myocarditis, myasthenia gravis, and myositis [13]. While ICIs have been touted to have an improved tolerability profile in comparison with chemotherapy, it is critical to be mindful of the wide range of tissues that can be affected by immune-mediated inflammation, with subsequent life-threatening organ failure. irAE are most common in the endocrine, skin, pulmonary, and gastrointestinal systems. However, treating physicians must be vigilant for less common immune diatheses, including neuropathic and myopathic syndromes such as the triad of myasthenia gravis, myocarditis, and myositis suffered by our patient. Here, we reviewed the principals of diagnosis and management of these rare but potentially fatal complications.

## Data Availability

The data supporting the findings of this study are available from the corresponding author upon reasonable request.

## Abbreviations

PD-L1: Programmed cell death ligand 1
irAE: Immune-related adverse event
NSCLC: Non-small cell lung cancer
SBRT: Stereotactic body radiation therapy
CK: Creatinine kinase
AST: Aspartate aminotransferase
ALT: Alanine aminotransferase
MRI: Magnetic resonance imaging
CT: Computed tomography
FDG: Fluorodeoxyglucose
INR: International normalized ratio
CMR: Cardiac MRI
MG: Myasthenia gravis
AchR: Anti-acetylcholine receptor antibody
MuSK: Anti-muscle-specific tyrosine kinase antibody
IVIG: Intravenous immunoglobulin
